# Influence of a guideline or an additional rapid strep test on antibiotic prescriptions for sore throat: the cluster randomized controlled trial of HALS (Hals und Antibiotika Leitlinien Strategien)

**DOI:** 10.1186/s12875-023-01987-w

**Published:** 2023-03-20

**Authors:** Hannelore Wächtler, Hanna Kaduszkiewicz, Oskar Kuhnert, Karolina Agata Malottki, Sonja Maaß, Jürgen Hedderich, Birgitt Wiese, Norbert Donner-Banzhoff, Julia Hansmann-Wiest

**Affiliations:** 1 Institute of General Practice, Kiel University, Michaelisstr. 5, 24105 Kiel, Germany; 2 Institute of Medical Informatics and Statistics, Kiel University, University Hospital Schleswig-Holstein, Campus Kiel, Arnold-Heller-Straße 3, Haus V40, 24105 Kiel, Germany; 3 IT Services Applications, Science & Laboratory, MHH Information Technology, Medizinische Hochschule Hannover (MHH), Carl-Neuberg-Str. 1, 30625 Hannover, Germany; 4 Department of General Practice / Family Medicine, University of Marburg, Karl-von-Frisch-Str. 4, 35043 Marburg, Germany

**Keywords:** Primary care, Sore throat, Pharyngitis, Group A beta-hemolytic streptococci, Rapid antigen detection test, Rapid strep test, Point-of-care test, Antibiotics, Guideline

## Abstract

**Background:**

Pharyngitis due to Group A beta-hemolytic streptococci (GAS) is seen as the main indication for antibiotics for sore throat. In primary care settings prescription rates are much higher than the prevalence of GAS. Recommendations in international guidelines differ considerably. A German guideline suggested to consider antibiotics for patients with Centor or McIsaac scores ≥ 3, first choice being penicillin V for 7 days, and recommended analgesics for all. We investigated, if the implementation of this guideline lowers the antibiotic prescription rate, and if a rapid antigen detection strep-test (RADT) in patients with scores ≥ 3 lowers the rate further.

**Methods:**

HALS was an open pragmatic parallel group three-arm cluster-randomized controlled trial. Primary care practices in Northern Germany were randomized into three groups: Guideline (GL-group), modified guideline with a RADT for scores ≥ 3 (GL-RADT-group) or usual care (UC-group). All practices were visited and instructed by the study team (outreach visits) and supplied with material according to their group. The practices were asked to recruit 11 consecutive patients ≥ 2 years with an acute sore throat and being at least moderately impaired. A study throat swab for GAS was taken in every patient. The antibiotic prescription rate at the first consultation was the primary outcome.

**Results:**

From October 2010 to March 2012, 68 general practitioners in 61 practices recruited 520 patients, 516 could be analyzed for the primary endpoint. Antibiotic prescription rates did not differ between groups (*p* = 0.162) and were about three times higher than the GAS rate: GL-group 97/187 patients (52%; GAS = 16%), GL-RADT-group 74/172 (43%; GAS = 16%) and UC-group 68/157 (43%; GAS = 14%). In the GL-RADT-group 55% of patients had scores ≥ 3 compared to 35% in GL-group (*p* < 0.001). After adjustment, in the GL-RADT-group the OR was 0.23 for getting an antibiotic compared to the GL-group (*p* = 0.010), even though 35 of 90 patients with a negative Strep-test got an antibiotic in the GL-RADT-group. The prescription rates per practice covered the full range from 0 to 100% in all groups.

**Conclusion:**

The scores proposed in the implemented guideline seem inappropriate to lower antibiotic prescriptions for sore throat, but better adherence of practitioners to negative RADTs should lead to fewer prescriptions.

**Trial registration:**

DRKS00013018, retrospectively registered 28.11.2017.

## Introduction

Pharyngitis due to Group A beta-hemolytic streptococci (GAS) is traditionally seen as the main indication for antibiotics for sore throat. Irrespective of the presence of GAS, the natural course of a sore throat in patients presenting in primary care is generally good, and it is only moderately shortened by antibiotics, slightly more in case of a throat swab positive for GAS [1]. Purulent complications are rare [2], and in industrialized countries non-purulent complications of GAS pharyngitis have become extremely rare [1, 3, 4]. On the other hand, bacteria increasingly develop resistances to antibiotics, depending on the frequency of their use and demanding an application as specific as possible [5–8].

Symptoms and signs of GAS pharyngitis are rather unspecific [9]. Several clinical prediction rules have been developed, the most widely established being the Centor score [10, 11] and the McIsaac score [12, 13]. The Centor score assigns one point each for the four criteria tonsillar exudates, swollen tender anterior cervical lymph nodes, lack of cough and history of fever. The McIsaac score also takes age into account (< 15 years one point added, > 45 years one point less). Another score, the feverPAIN score aims to predict the probability of pharyngitis by group A, C or G streptococci [14, 15].

The culture of a throat swab for GAS is time consuming, but still the gold standard for the diagnosis of GAS pharyngitis. Rapid strep tests (RADTs = Rapid antigen detection tests for GAS) as point-of-care tests deliver a quick result in the office [16, 17]. But, in our preceding observational study German general practitioners (GPs) performed a RADT only in about 3% of patients presenting with sore throat [18].

In high-income countries the mean GAS prevalence in patients with pharyngitis is about 25% [19], whereas reported antibiotic prescription rates are about two to three times higher [20, 21, 22].

Few trials have studied the influence of diagnostic tools like clinical scores and/or RADTs on antibiotic prescriptions and patient outcomes in daily practice. While the sole use of scores failed to reduce prescriptions [23, 24], RADTs with or without scores seemed to reduce prescribing rates [24, 25]. A British trial run at about the same time as our study found a reduction of dispensed antibiotic treatments and some benefit in patient outcome for the application of their new feverPAIN score as well as for the combination of score and RADT [14]. A recent Cochrane Review on RADTs for sore throat, including five randomized controlled trials (RCTs), among them the trials mentioned above [14, 24, 25], showed a reduction of antibiotic prescriptions in the rapid test groups versus management on clinical grounds, mostly using scores [26].

Recommendations in international guidelines differ considerably. They cover the full range from a pure clinical approach to demanding a positive rapid test and/or culture for GAS before prescribing an antibiotic [27]. The guideline “Sore Throat” of the German College of General Practitioners and Family Physicians (DEGAM) was first published in 2009 [28, 29], and has been updated in 2020 [30, 31]. In our study we used the guideline from 2009. It suggests to use the Centor or McIsaac score, to consider antibiotics for more severely affected patients with scores ≥ 3 to be taken immediately or delayed (meaning only in case of persistent or worsening illness), first choice being penicillin V for 7 days, and it recommends analgesics for all. GPs should inform the patient about the favourable natural course of the illness, and the modest effect of antibiotics. It is up to the GP to take a throat swab for RADT or culture for GAS.

We hypothesized that the active implementation of the guideline would lower the prescription rate, and that an additional RADT in all patients with scores ≥ 3 and suggesting antibiotic prescriptions only to patients with a positive test for GAS would lower the rate further. We expected that both strategies would have no effect on patient outcomes.

## Methods

HALS was an open, three-armed, cluster-randomized, controlled pragmatic trial (cRCT).

### Trial recruitment

At continuous medical education meetings in northern Germany (Schleswig–Holstein), we asked general practitioners and specialists in internal medicine working in general practice (all named “GPs”) to participate in the study (convenience sample). A maximum of 2 GPs per practice could take part.

### Randomization into three study arms

The practices were randomized by Jürgen Hedderich with the computer program BiAS [32] to one of three groups (parallel group trial design, allocation ratio 1:1:1):

- Guideline group (GL-group): The GPs were instructed to follow the DEGAM guideline 2009 as outlined in the introduction.
- Guideline and targeted rapid antigen detection test group (GL-RADT-group): GPs were to proceed as in the GL-group, but should take a throat swab for a RADT in all patients with a Centor or McIsaac score ≥ 3, and offer an immediate or delayed antibiotic therapy only to patients with a positive RADT or, in case of a negative RADT, if GAS could be shown in a culture initiated by the practice, independent of the study throat swab (see below). We chose the Alere™ TestPack + Plus Strep A test with OBC, produced by Inverness Medical Innovations until 2010, because of good in vitro performance and ease of use [33].
- Usual care group (UC-group): GPs should follow their normal routine.

The guidelines in the GL- and GL-RADT-group provided recommendations, but ultimately the GPs themselves decided on the medical treatment.

### Implementation

The initiator of the study, an experienced GP, and four medical doctoral students in the study team performed the outreach visits. The doctoral students were trained by the GP in communicating the contents, objectives and performance of the study to the practices. After randomization each practice was visited by a member of the outreach visit team, instructed according to the allocated group and supplied with the corresponding material in paper format. We introduced a printed version of the DEGAM-guideline to the intervention groups (GL-group and GL-RADT-group). All practices were instructed in the technique of taking a study throat swab, and the practices of the GL-RADT-group to perform the rapid test.

### Recruitment of patients

We asked the practices to include 11 consecutive patients aged ≥ 2 years at their first consultation because of a sore throat suggesting an infection, lasting less than two weeks and being at least moderately impairing.

The practices should exclude patients with only mild impairment for whom the guideline did not recommend antibiotics. Other exclusion criteria were another disease demanding antibiotics, scarlet fever, quinsy, a personal or family history of acute rheumatic fever, a consuming disease or immunosuppression, or insufficient knowledge of the German language.

### Data collection

#### Practice questionnaire

Each practice documented size of the practice measured as number of patients per quarter of the year, numbers of GPs in the practice, and location of the practice (rural or urban), age and gender of participating GPs, undergraduate medical education activity, and specialization for natural medicine.

#### Study throat swab for GAS

The practices were instructed to take a study throat swab in each patient. With direct vision and good lighting, the cotton swab should be passed over both tonsils and the posterior pharyngeal wall in a rolling and rubbing motion, preferably without touching the tongue, uvula or buccal mucosa [34]. The swabs were sent to the Institute for Infection Medicine at the University Medical Center Schleswig–Holstein, Campus Kiel. We supplied the practices with Copan Transystem™ units with Amies agar gel medium. In the laboratory, the specimens were cultivated on a Columbia blood plate (Columbia blood agar with sheep blood from Oxoid) at 36°Celsius for 24 h. Finally, from subcultures of suspicious colonies on the blood agar, latex agglutination confirmed group A streptococci (Prolex® Streptococcus latex reagent from Pro-Lab Diagnostics). The results were sent to the study center only. After termination of the data collection, the results were given to the practices as feedback information.

#### GP case report form

Immediately after the consultation the GPs filled in a one-sided form with the date of the consultation, the patient’s age and gender, data detailing the disease (previous duration of sore throat, general impairment by the illness [moderate or severe], severity of the sore throat [mild, moderate or severe], fever ≥ 38° C [actual or in history]), suspicion of pharyngitis caused by GAS, laboratory tests (throat swab for RADT or culture, apart from the study throat swab; others) and therapy (prescription of an antibiotic, to be taken immediately or delayed, kind of antibiotic and duration of therapy, other recommendations). GPs in the intervention groups were also asked to document the Centor and McIsaac score. The form was sent via fax to the study center.

#### Patient questionnaire concerning the actual consultation

Still in the office, the patients answered questions on a two-sided questionnaire about age and gender, sociological data (degree of education, actual job, number of persons in the household [overall and persons < 18 years]), reasons for consulting the GP on a 4-point Likert scale (severity of symptoms, wish to learn a diagnosis, the prognosis or to be informed about treatment options, desire to get a drug for relief of symptoms or desire to get an antibiotic prescription, need of a sick note, wish to be referred to a specialist, and satisfaction with the consultation). The patients then sent the questionnaire directly to the study center by mail.

#### Journal

The patients were asked to keep a journal every night for ten days. This contained questions covering feeling of discomfort, severity of sore throat, fever, impairment in daily activities, and absence from kindergarten, school or work, and the treatment taken (an analgesic, an antibiotic or others).

#### List of patients not included

GPs were asked to document the date of the consultation, and the reasons for non-participation of patients not included in the study in a prepared list.

### Outcomes

No changes in planned outcome measures were made after the start of the trial.

#### Primary outcome

The primary outcome was the prescription rate of antibiotics at the first consultation as documented by the GPs. This included both antibiotics to be taken immediately and delayed prescriptions.

#### Predefined secondary outcomes

Secondary outcomes included type of antibiotics prescribed and duration of therapy, recommendation of analgesics, diagnostic tests performed and their results, all as documented by the GPs, the clinical course of the illness, including the consumption of antibiotics or analgesics as documented in the patients’ journals, and the rate of GAS.

### Sample size considerations

In our preceding epidemiologic study 41% of all patients with a sore throat as their main complaint received an antibiotic prescription at their first consultation, and the prescription rate was significantly associated with the degree of impairment by the illness [18]. By excluding patients with only mild impairment (19% of patients in the observational study, of whom 18% got an antibiotic prescription) we expected a prescription rate of 45% in the UC-group. A two group Chi^2^ test with 0.017 two sided significance level (α = 0.05, adjusted for multiplicity) will have 80% power to detect a reduction to 25% in this rate when the sample size in each group is *n* = 128. Cluster randomized clinical trial cRCT-adjustment with respect to an intra-cluster correlation coefficient (ICC = 0.05, assumed), and a cluster size of k = 10 patients per practice leads to 190 patients in 19 practices per study group. Assuming a dropout rate of practices of 20% a total of 75 practices would have to be recruited (25 practices per group).

### Data analysis

Data were analyzed with SPSS and STATA. The characteristics of practices, GPs and patients were analyzed descriptively. Study arms were compared with the Chi^2^-test for nominally and ordinal scaled data, and using univariate analysis of variance for metric variables. The four-point Likert scale for patient self-assessments and their expectations of the consultation was dichotomized in (strongly agree + agree) versus (disagree + strongly disagree).

The primary outcome measure, antibiotic prescription rate, was compared pairwise between study arms by 95% confidence intervals (exact binomial distribution). The comparison between all three study arms was carried out with the Chi^2^ test. An adjustment for a possible cluster (practice) effect was evaluated in a logistic regression analysis with practice-id as a random effect. Scatterplots were created to illustrate the heterogeneity of antibiotic prescriptions and GAS rates.

Secondary outcome measures were analyzed descriptively. The Chi^2^-test was used to compare groups with regard to the type of antibiotics prescribed, the duration of therapy when penicillin V was prescribed, and the recommendation of analgesics. It was also used to compare the resolution of discomfort and sore throat, and the patients’ adherence to antibiotic and analgesic therapy as documented in their journals. The guideline proposes to consider antibiotics for patients with Scores ≥ 3. So, despite the different scale widths of the Centor and McIsaac score (Centor 0 to 4 points, McIsaac -1 to 5 points), the score values were uniformly dichotomised into ≤ 2 and ≥ 3 points. In case of two different doctor's statements regarding the Centor and McIsaac score, the higher value was used. For the Centor and McIsaac scores ≤ 2 and ≥ 3 as well as for the RADT sensitivity, specificity, and positive predictive values were calculated.

In order to adjust for imbalance between study arms, mixed effects logistic regression analyses were performed with the target variables antibiotic prescription (models 1 and 2) and advice to take analgesics (model 3) and the following control variables: study-group, GP gender, age, activity in undergraduate medical education, and specialization in natural medicine, patient gender, age, severity of sore throat and general impairment, fever, wish to get a relieving remedy, and wish to get an antibiotic. As random effect the practice-ID was included in the models. In model 2, the variables used in model 1 were supplemented by the Centor or McIsaac score (≤ 2 or ≥ 3). However, this analysis was only possible for the intervention groups because no scores were determined in the UC-group. The results of the multivariate regression analyses are presented as odds ratios with 95% confidence intervals.

## Results

**Fig. 1:**
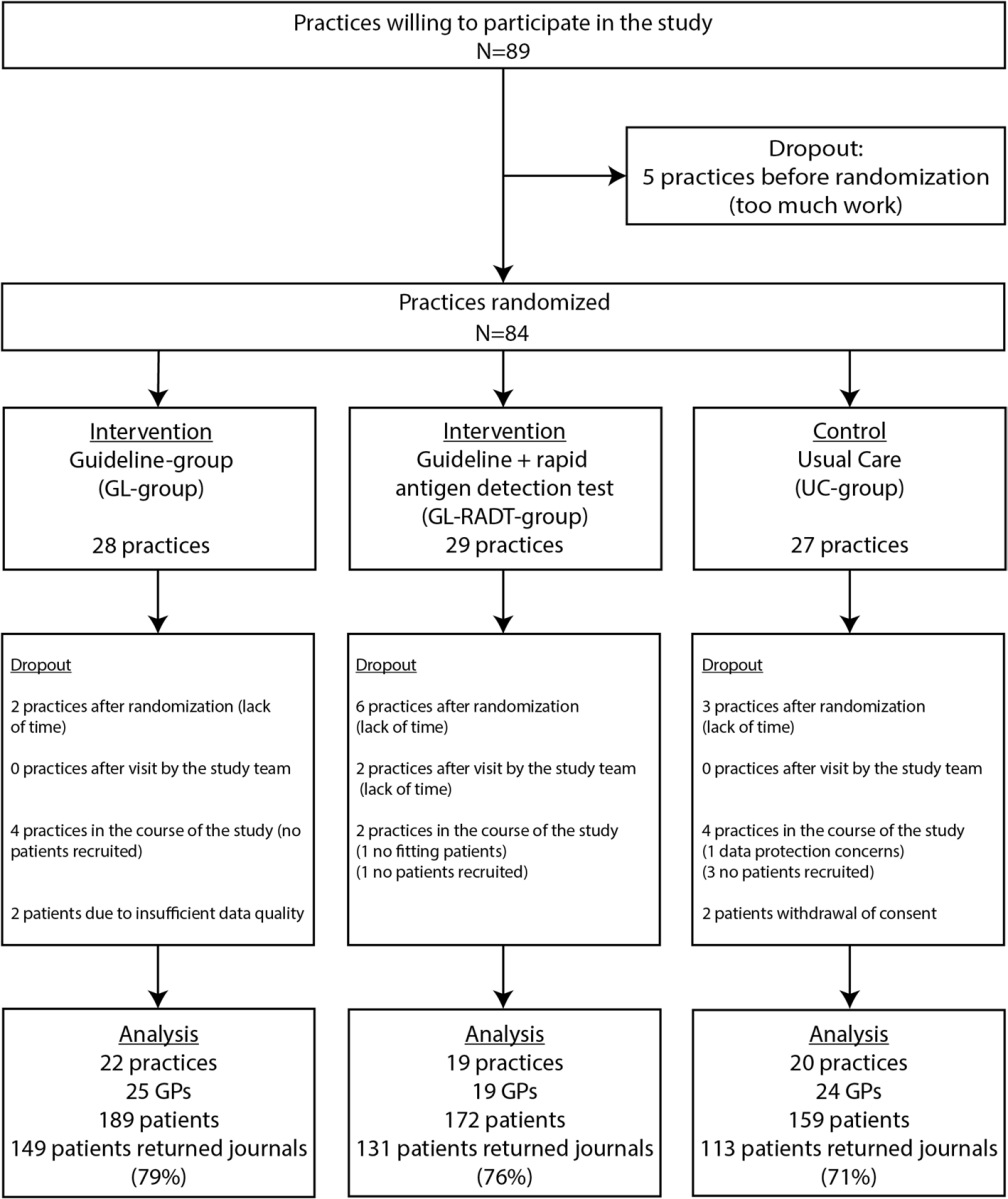
Flow chart of practices, GPs and patients

From October 15^th^ 2010 until March 30^th^ 2012, 68 GPs in 61 practices recruited 524 patients with a sore throat as main complaint. The data of 520 patients could be analyzed (Fig. 1).

### Group characteristics

There were no statistically significant differences between the study arms regarding the characteristics of practices (number of patients per quarter, number of GPs working in the practice, locality of the practice), GPs (sex, age, specialization for natural medicine, activity in undergraduate medical education), and patients (sex, age, degree of education, number of persons in the household, and number of persons under 18 years in the household).

The GPs were on average 50 years old, and 66% male. 65% were active in undergraduate medical education, and 12% had a specialization for natural medicine. 56% of the practices (34/61) recruited the maximally allowed number of 11 patients. Patients were on average 33 years old (modal 20, median 30), and 64% female (Table 1).

**Table 1:** Selected characteristics of practices, GPs and patients

	**Guideline** **(GL-group)**	**Guideline + RADT** **(GL-RADT-group)**	**Usual care** **(UC-group)**	**Total**	*p*-value*
**Practices**	***N* = 22**	***N* = 19**	***N* = 20**	***N* = 61**	
Patients per quarter^§^					
< 500	2 (9)	0 (0)	2 (10)	4 (7)	
500—999	12 (55)	9 (47)	4 (20)	25 (41)	0.103
1.000 – 1.499	5 (23)	4 (21)	9 (45)	18 (30)	
≥ 1.500	3 (14)	6 (32)	5 (25)	14 (23)	
Mean Number ± SD of GPs working in the practice	1.9 ± 0.9	2.0 ± 1.0	2.0 ± 1.1	2.0 ± 1	0.883**
Locality of practice					
> 50.000 residents	9 (41)	7 (37)	9 (45)	25 (41)	
10.000 – 50.000 residents	5 (23)	5 (26)	5 (25)	15 (25)	0.983
< 10.000 residents (rural)	8 (36)	7 (37)	6 (30)	21 (34)	
**GPs**	***N* = 25**	***N* = 19**	***N* = 24**	***N* = 68**	
Female	8 (32)	5 (26)	10 (42)	23 (34)	0.556
Mean age [years] ± SD	50 ± 11	54 ± 8	47 ± 7	50 ± 9	0.072**
Undergraduate medical education activity	16 (64)	12 (63)	16 (67)	44 (65)	0.968
Specialized for natural medicine	3 (12)	3 (16)	2 (8)	8 (12)	0.863
**Patients**	***N* = 189**	***N* = 172**	***N* = 159**	***N* = 520**	
Female	129 (68)	110 (64)	94 (60)	333 (64)	0.237
Mean age [years] ± SD	35 ± 16	32 ± 16	33 ± 13	33 ± 15	0.163**
Modal value [years]	19	20	19***	20	
Number of persons in the household:	***N* = 167**	***N* = 160**	***N* = 148**	***N* = 475**	
- one	24 (14)	22 (14)	26 (18)	72 (15)	
- two	46 (32)	54 (34)	46 (31)	146 (32)	0.360
- three	48 (27)	26 (16)	36 (24)	110 (23)	
- four	34 (19)	41 (26)	27 (18)	102 (21)	
- five and more	15 (9)	17 (11)	13 (9)	45 (9)	
Number of persons aged < 18 years in the household:	***N* = 172**	***N* = 160**	***N* = 145**	***N* = 477**	
- zero	96 (56)	89 (56)	82 (57)	267 (56)	0.990
- one	35 (20)	31 (19)	29 (20)	95 (20)	
- two	29 (17)	30 (19)	27 (19)	86 (18)	
- three and more	12 (7)	10 (6)	7 (5)	29 (6)	

Figures are numbers (percentages), unless stated otherwise. SD standard deviation; *Chi^2^ test unless indicated; **Univariate analysis of variance; ^§^irrespective of how many times the patients consult the practice per quarter they are counted once; ***2 modes exist, the smallest value is shown

### Patients’ self-assessment and expectations, clinical assessment by the GPs and GAS-results in throat swabs

Eighty eight percent of patients complained of severe discomfort, 87% desired a relief of discomfort (symptomatic treatment) with the highest rate in the GL-RADT-group. However, only 32% of all patients intended to receive an antibiotic, without differences between study arms.

Seventy three percent of all patients had a sore throat for 1 to 3 days before seeing the GP. The mean duration of sore throat until consultation was 3 days without relevant differences between groups.

The GPs documented a severe sore throat in 61% of patients, a severe general impairment in 51% of patients, and fever in 34%. In the GL-RADT-group slightly more patients were severely impaired, had a severe sore throat, and fever compared to the other study groups. Centor or McIsaac scores of ≥ 3 were documented in the GL-RADT in 55%, and in the GL-group in 35% of patients (*p* ≤ 0.001). Scores were not documented in the UC-group in order not to influence the GPs. Analogous to the Centor or McIsaac score of ≥ 3, more physicians in the GL-RADT group (51%) expressed suspicion of GAS pharyngitis at the end of their clinical evaluation than in the GL-group and UC-group (44% and 35%).

In the study throat swabs the prevalence of GAS (15%) was similar in all three groups (Table 2).

**Table 2:** Patient self-assessment and expectation, clinical assessment by the GPs and GAS-results in study throat swabs

	**Guideline** **(GL-group)** ***N* = 189**	**Guideline + RADT** **(GL-RADT-group)** ***N* = 172**	**Usual care** **(UC-group)** ***N* = 159**	**Total** ***N* = 520**	*p*-value*
Patients’ self-assessment and expectations***					
Severe discomfort	148/175 (85)	149/162 (92)	130/150 (87)	427/487 (88)	0.107
Wish for a relieving remedy	154/178 (87)	153/165 (93)	124/150 (83)	431/493 (87)	**0.024**
Wish to get an antibiotic	62/178 (35)	52/163 (32)	42/146 (29)	156/486 (32)	0.486
**Clinical assessment by the GPs**					
Days of sore throat until consultation [Mean ± SD]	3.3 ± 2.6	2.9 ± 1.9	2.7 ± 2.3	3.0 ± 2.3	0.030**
1–3 days	127/182 (70)	123/168 (73)	119/155 (77)	369/505 (73)	0.474
4–7 days	46/182 (25)	41/168 (24)	30/155 (19)	117/505 (23)	
8–14 days	9/182 (5)	4/168 (2)	6/155 (4)	19/505 (4)	
Severity of sore throat					
mild	12/180 (7)	9/169 (5)	5/154 (3)	26/503 (5)	0.312
moderate	60/180 (33)	49/169 (29)	59/154 (38)	168/503 (33)	
severe	108/180 (60)	111/169 (66)	90/154 (58)	309/503 (61)	
General impairment by the illness					
moderate	93/182 (51)	74/164 (45)	78/152 (51)	245/498 (49)	0.444
severe	89/182 (49)	90/164 (55)	74/152 (49)	253/498 (51)	
Fever ≥ 38 °C					
actual or in history	52/189 (28)	68/172 (40)	53/159 (33)	173/520 (34)	0.167
not reported	25/189 (13)	22/172 (13)	18/159 (11)	65/520 (13)	
Centor- or McIsaac Score					
≤ 2	101/189 (53)	69/172 (40)	data not	170/361 (47)	** ≤ 0.001**
≥ 3	67/189 (35)	95/172 (55)	collected	162/361 (45)	
not reported	21/189 (11)	8/172 (5)		29/361 (8)	
Suspicion of GAS pharyngitis by GP	77/174 (44)	82/161 (51)	54/154 (35)	213/489 (44)	**0.017**
**Study throat swab**					
Cultures positive for GAS	29/184 (16)	26/161 (16)	21/154 (14)	76/499 (15)	0.799

Figures are numbers (percentages), unless stated otherwise. SD standard deviation, *Chi^2^ test unless indicated, **Univariate analysis of variance, ***dichotomized 4-point Likert scale: (strongly agree + agree) vs. (disagree + strongly disagree)

### Primary outcome: Prescription rate of antibiotics to be taken immediately or only in case of worsening illness

Overall 46% (239/516) of patients received an antibiotic prescription, 5% (13/239) of the delayed type. The unadjusted analysis without clustering taken into account showed no significant differences between groups. Though the data revealed a trend for more prescriptions in the GL-group (52%) compared to the GL-RADT-group (43%) or the UC-group (43%), correction for clusters (GP practice) in the regression analysis confirmed the absence of significant group differences (Table 3).

**Table 3:** Prescription of antibiotics (AB)

	**Descriptive Statistics**				Mixed effects, cluster corrected for GP practice, logistic regression with AB prescription as outcome and study group as influencing parameter*		
	**Number of prescriptions (%)**	**Number of prescriptions 95% Confidence interval**	p**	Delayed prescriptions in all AB prescriptions (%)	**Odds Ratio**	**95% Conf. Interval**	**p**
**GL-group**	97/187 (52)	45% – 59%	0,162	6/97 (6)	2.32	0.81 – 6.66	0.118
**GL-RADT-group**	74/172 (43)	36% – 51%		5/74 (7)	1.05	0.36 – 3.04	0.934
**UC-group**	68/157 (43)	35% – 51%		2/68 (3)	1 (reference)		
Total	239/516 (46)			13/239 (5)			

^*^N = 516 (2 cases missing in GL- and UC-group each); ICC (95% CI): 0.37 (0.23 – 0.54), **Chi^2^-test comparing the three arms

Overall, the antibiotic prescription rate was approximately three times higher than the GAS rate. Only 24% of the patients receiving an antibiotic prescription had a positive culture for GAS (26% in the GL-RADT-group, as well as the UC-group, 21% in the GL-group).

**Fig. 2:**
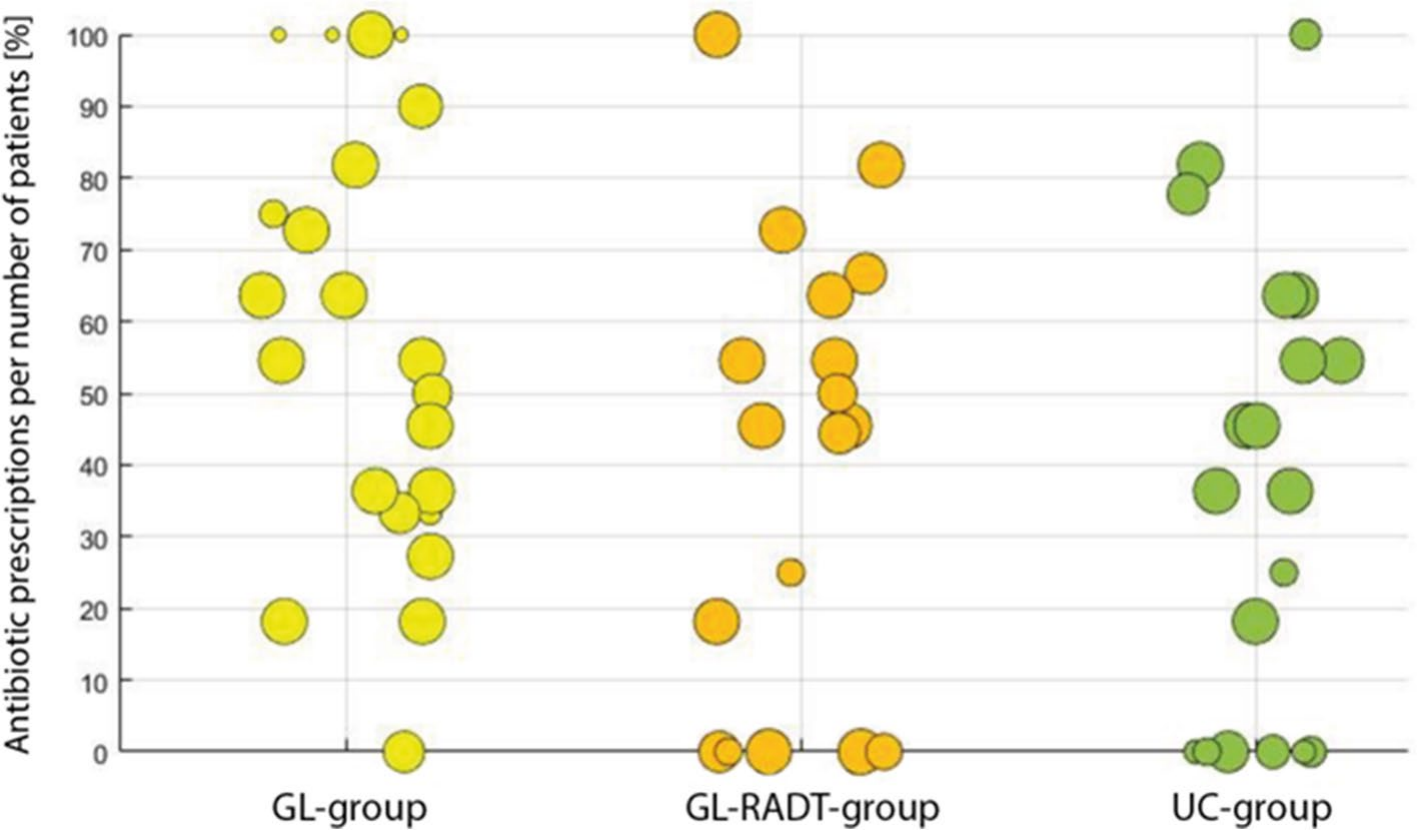
Scatterplot with antibiotic prescription rates per GP-practice. Each practice is one circle. The size of the circles is determined by the numbers of patients (k = 1–11) per practice

**Fig. 3:**
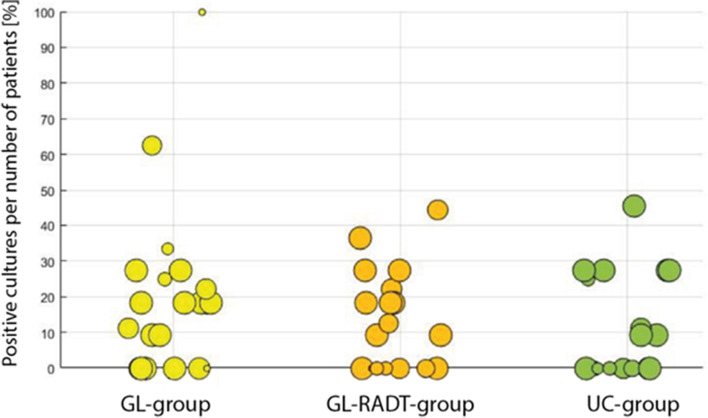
Scatterplot with GAS-prevalence rates in % per GP-practice. Each practice is one circle. The size of the circles is determined by the numbers of study throat swabs (k = 1–11) per practice

The prescribing behavior across practices was very heterogeneous, with a range of antibiotic prescriptions from 0 to 100% in all 3 groups (Fig. 2). The rate of positive cultures for GAS (mean value 15%) varied considerably between practices, too (Fig. 3).

### Factors associated with antibiotic prescription

We performed two multivariable logistic regression analyses with antibiotic prescription as outcome, corrected for cluster and including 12 variables, among them the study group (Table 4).

**Table 4:** Mixed effects logistic regression analyses with the outcomes antibiotic prescription and advice to take analgesics

	**Model 1** **Antibiotic prescription, N = 458**			**Model 2** **Antibiotic prescription,** **only intervention arms, N = 320**			**Model 3** **Advice to take analgesics, N = 451**		
	**OR**	**95% CI**	***p*-value**	**OR**	**95% CI**	***p*-value**	**OR**	**95% CI**	***p*-value**
**Arm** UC	1			–-	–-	–-	1		
GL	2.35	0.71–7.76	0.161	1			6.17	1.40–27.13	**0.016**
GL-RADT	0.65	0.19–2.20	0.488	0.23	0.08–0.71	**0.010**	35.39	7.32–171.17	** < 0.001**
**GP gender** f vs. m	1.09	0.40–3.98	0.861	1.13	0.37–3.46	0.833	1.47	0.42–5.10	0.543
**GP age** (years)	1.05	0.99–1.11	0.131	1.02	0.96–1.09	0.526	0.90	0.83–0.97	**0.006**
**Teaching GP**	0.98	0.36–2.66	0.962	0.49	0.17–1.39	0.178	2.12	0.60–7.51	0.243
**Specialised for natural medicine**	0.11	0.21–0.58	**0.009**	0.15	0.03–0.85	**0.032**	0.58	0.09–3.76	0.566
**Patient gender f vs. m**	0.92	0.51–1.64	0.768	0.80	0.39–1.66	0.547	1.12	0.62–2.04	0.710
**Patient age** (years)	1.00	0.98–1.02	0.825	1.02	1.00–1.04	0.117	0.99	0.97–1.01	0.446
**Severity of sore throat**									
light	1			1			1		
moderate	0.81	0.20–3.20	0.762	0.66	0.11–3.81	0.640	0.88	0.22–3.53	0.860
severe	2.07	0.51–8.41	0.309	1.46	0.24–8.75	0.680	1.31	0.32–5.49	0.706
**General impairment by the illness**									
severe vs. moderate	1.37	0.68–2.75	0.372	0.95	0.39–2.34	0.919	1.33	0.62–2.85	0.457
**Fever yes vs. no**	2.60	1.34–5.01	**0.004**	1.35	0.57–3.19	0.491	0.97	0.48–1.96	0.929
**Wish to get a relieving remedy**	7.31	2.58–20.72	** < 0.001**	2.92	0.82–10.46	0.100	1.37	0.55–3.45	0.499
**Wish to get an antibiotic**	10.11	5.17–19.78	** < 0.001**	12.18	5.17–28.70	** < 0.001**	0.62	0.32–1.23	0.171
**Centor or McIsaac score**									
≤ 2	–-	–-	–-	1			–-	–-	–-
≥ 3	–-	–-	–-	13.03	5.11–33.23	** < 0.001**	–-	–-	–-
not done	–-	–-	–-	2.75	0.50–15.11	0.245	–-	–-	–-

All models are cluster-corrected for GP-practice

#### Model 1

In model 1 all three groups were considered. The patient’s wish to get an antibiotic showed the strongest association with receiving a prescription (OR 10.11; *p* < 0.001), followed by the patient’s wish to get a relieving remedy (OR 7.31; *p* < 0.001). Fever was weakly associated with an antibiotic prescription (OR 2.60; *p* = 0.004) whereas a GP’s specialization for natural medicine made a prescription less likely (OR 0.11; *p* = 0.009).

Neither the study group, nor the severity of sore throat, nor the degree of general impairment showed any association with an antibiotic prescription. Further there was no association with sex and age of patients and GPs, or with the GP being active in undergraduate medical education.

#### Model 2

The Centor or McIsaac scores were added to the variables. Since the scores were only obtained in the intervention groups GL, and GL-RADT, this analysis was performed in these groups only. 72% of patients with scores ≥ 3 got an antibiotic compared to 26% of patients with scores ≤ 2 (*p* < 0.001). The number of patients with scores ≥ 3 was significantly higher in the GL-RADT-group than in the GL-group. In model 2 scores ≥ 3 showed the strongest association with getting an antibiotic prescription (OR 13.03; *p* < 0.001). Adjustment for the scores made a prescription less likely in the GL-RADT-group compared to the GL- group (OR 0.23; *p* 0.010).

### Scores, rapid tests and antibiotic prescription rates

Scores ≥ 3 versus scores ≤ 2 had a sensitivity of about 67% and a specificity of about 55%, resulting in a positive predictive value of about 23% without differences between Centor score and McIsaac score. The test quality criteria of the RADT for all patients with RADT and culture test results (N = 117, GAS-prevalence 17%) were as follows: sensitivity 65%, specificity 85%, positive predictive value 46%.

In the GL-RADT-group, a RADT should have been performed in the 95 patients with scores ≥ 3 of 172 patients in this study group (55%). The GPs however reported test results in 126 patients (74%): in 80 of the 95 patients with scores ≥ 3, in 37 of 69 patients with scores ≤ 2 and in 2 of 8 patients without a documented score. All 29 patients with a positive RADT and 35 of the 90 patients (39%) with a negative RADT received an antibiotic. Antibiotic prescriptions in patients with a negative RADT depended on the value of the Centor or McIsaac score: in the group with a score ≥ 3 the prescription rate was 50%, in the group with a score ≤ 2 the rate was 23%. The RADT seems to have contributed to the antibiotic prescription rate of 43% being lower than the rate of Centor or McIsaac scores ≥ 3 (55%) in the GL-RADT-group (Table 5).

**Table 5:** Antibiotic prescriptions according to Centor/McIsaac criteria and RADT results in the GL-RADT-group

Guideline and targeted rapid antigen detection group (GL-RADT) group: 172 patients																			
≥ 3 Centor or McIsaac criteria 95 patients (55%)						≤ 2 Centor or McIsaac criteria 69 patients (40%)						no score documented 8 patients (5%)							
RADT 80 patients				no RADT 15 patients		RADT 37 patients				no RADT 32 patients		RADT 2 patients						no RADT 6 patients	
+ 26		- 54				+ 2		- 35				+ 1			- 1				
AB	no AB	AB	no AB	AB	no AB	AB	no AB	AB	no AB	AB	no AB	AB	no AB	AB		no AB	AB		no AB
26	0	27	27	7	8	2	0	8	27	0	32	1	0	0		1	3		3
65% AB prescription rate						14% AB prescription rate						50% AB prescription rate							

*AB* Antibiotics, + positive RADT result, - negative RADT result

In the GL-group, the GPs documented scores ≥ 3 in only 67 out of 189 patients (35%) and prescribed an antibiotic to 56 of these 67 patients (84%), while the overall antibiotic prescription rate was 52% in the GL-group (Table 6).

**Table 6:** Antibiotic prescriptions according to Centor/McIsaac criteria in the GL-group

Guideline (GL)-group: 189 patients					
≥ 3 Centor or McIsaac criteria 67 patients (35%)		≤ 2 Centor or McIsaac criteria 101 patients (53%)		no score documented 21 patients (11%)	
AB	no AB	AB	no AB	AB	no AB
56	11	34	67	7	12
84% AB prescription rate		34% AB prescription rate		33% AB prescription rate	

*AB* Antibiotics

In the UC- and GL-group physicians performed a RADT on their own initiative in only 14 of 348 patients (4%) without reporting the results in the study documents.

### Predefined secondary outcomes: Rate of penicillin V prescriptions and duration of penicillin V therapy (7 days versus 10 days) as documented by the GPs

**Fig. 4:**
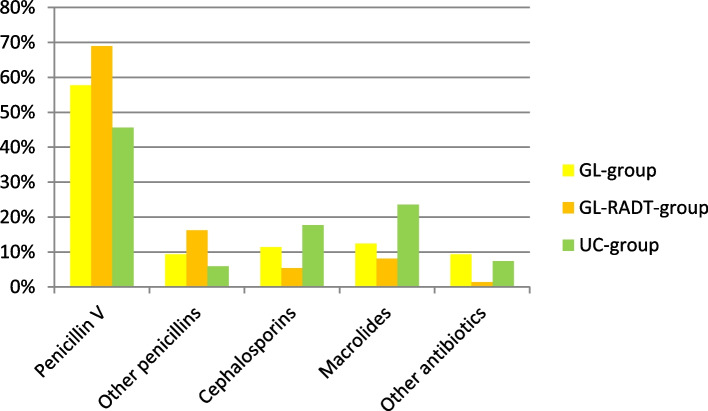
Proportion of different antibiotics in prescriptions. GL-group *N* = 97, GL-RADT-group *N* = 73, UC-group *N* = 68

**Fig. 5:**
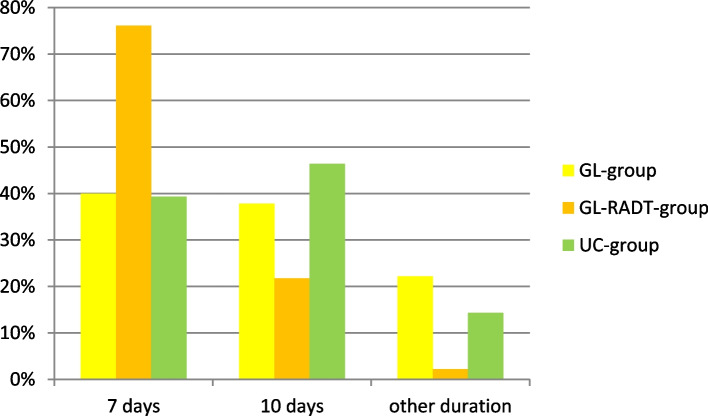
Duration of treatment with penicillin V. GL-group *N* = 45, GL-RADT-group *N* = 46, UC-group *N* = 28

The rate of penicillin V prescriptions compared to other antibiotics was higher in the intervention groups (GL-RADT- group 70%, GL-group 58%, UC-group 46%, *p* = 0.014). GPs in the rapid test group showed the best adherence to a 7 days course (GL-RADT-group 76%, GL-group 40%, UC-group 39%, *p* = 0.001) (Figs. 4 and 5).

### Recommendation of analgesics as documented by the GPs

In both intervention groups, the GPs recommended analgesics more often than in the UC group, with the highest rate in the rapid test group (GL-RADT-group 62%, GL-group 45%, UC-group 23%, *p* < 0.001).

A third cluster-corrected logistic regression analysis including the same independent variables as model 1, but with the outcome “advice to take analgesics” confirmed these findings with an OR of 35.39 for the GL-RADT group versus the UC-group (*p* < 0.001) and an OR of 6.17 for the GL-group versus the UC-group (*p* = 0.016). Overall, younger GPs were more likely to recommend analgesics (Table 4, model 3).

### Resolution of discomfort and sore throat and patients’ adherence as documented in the journal

**Fig. 6:**
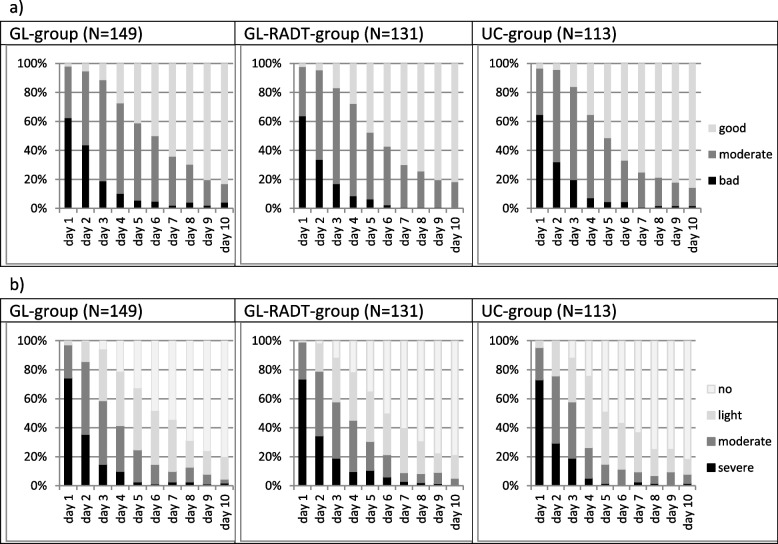
Patient reports in the journals: general discomfort (**a**), course of the sore throat (**b**)

Three hundred ninety three of 520 patients (76%) returned their journal. On the day of consultation (day 1) 63% complained of severe discomfort, on day three these were 18%. Sore throat symptoms also declined rapidly: On day one 74% of patients reported severe sore throat, on day three these had dropped to 16%. On day 10, about 80% of the patients felt good, and no longer had a sore throat. 6% of all the patients still complained of an at least moderate sore throat at this point (Figs. 6a and b). There were no significant differences between groups. Our data do not provide reliable information on complications or side effects of antibiotics.

Patients adhered well to antibiotic prescriptions, and recommendations of analgesics. If antibiotics were prescribed and taken (*N* = 181), 54% of patients adhered to the prescribed duration. 20% stopped earlier, and 26% took them longer. Four patients reported to have stopped antibiotics because of side effects. Patients who had got the recommendation to take analgesics documented a mean intake of 2–3 days. There were no significant differences between groups, except for taking analgesics for about half a day longer in the GL-RADT-group.

## Discussion

In unadjusted analyses, neither the active implementation of a guideline recommending the Centor or McIsaac score nor the addition of a targeted rapid antigen test reduced antibiotic prescriptions at a first consultation for sore throat in patients being at least moderately impaired by their illness. The prescription rate of 46% overall was about 3 times higher than the GAS rate of 15%. Only 24% of the patients receiving an antibiotic prescription had a positive culture for GAS*.* Delayed prescriptions are unusual in Germany.

The prescription rates concur with ranges reported in other studies [14, 25]. They all exceed the range of 0 to 20% proposed as quality indicator for sore throat or tonsillitis [35].

Regarding the GL-group and the UC-group, our findings confirm the results of former studies [23, 24] that clinical scores will not lower antibiotic prescriptions in patients with sore throat. We cannot exclude that some GPs in the control group might also have used scores. About two thirds of the participating GPs were active in undergraduate medical education, and therefore may be familiar with guidelines. As the guideline had been published shortly before the start of this study, GPs in the control group might have been exposed to it. Thus contamination cannot be excluded. On the other hand, the prescription rate of 43% in the UC-group corresponds quite well with the rate of 41% at the first consultation in our preceding observational study, including all patients presenting with a sore throat. This observational study was conducted previous to the publication of the guideline (inclusion of re-consultations had resulted in a total prescription rate of 43%) [18]. In the current study there was no association between GP activity in undergraduate medical education and antibiotic prescription rate.

Following the clinical assessment, GPs suspected a GAS-pharyngitis even more often in the intervention groups than in the UC-group (GL-group 44% and GL-RADT-group 51% versus 35% in the UC-group), despite a similar GAS-rate of about 15% in all groups. The diagnostic properties of the Centor and McIsaac scores ≤ 2 and ≥ 3 in our sample with a positive predictive value of 23% are in the range of the results found by a systematic review for the Centor score in general practice, confirming that the lower the pretest probability of GAS pharyngitis, the higher the overestimate via scores [10].

There was, however, a significant imbalance in our sample with scores ≥ 3 being more frequent in the GL-RADT-group than in the GL-group. This may be due to some selective recruitment of patients. The regression analysis comparing the intervention groups only, showed that scores ≥ 3 were the strongest predictor for an antibiotic prescription (OR 13.03, p < 0.001) and adjustment led to fewer antibiotic prescriptions in the GL-RADT-group (OR 0.23; p 0.010). This suggests an influence of the RADT.

The British RCT PRISM was run overlapping with our trial and compared their new FeverPAIN score to predict the presence of A, C and G streptococci (score group) with a combination of their score and targeted use of RADTs (RADT group) versus a strategy of “Delayed prescription” for all as control [14]. 37% of the patients in the score group and 35% in the RADT group used antibiotics compared to 46% in the “Delayed prescription” group. The authors do not report score values nor the number of RADTs applied nor the results or GP adherence to the results. The antibiotic consumption rates in the intervention groups are probably much higher than the rate of streptococcal infections to be assumed.

The Spanish cRCT conducted in primary care was published during our trial. It included adults with at least one criterion of the Centor score, and compared the effect of rapid testing in all patients to usual care without RADT [25]. The score values were determined by the study team. The GAS rate of 16.7% was similar to ours. The antibiotic prescription rate of 44% in the RADT-group was lower than in the control group (64%).

In our trial all patients with a positive rapid test were prescribed an antibiotic, but also 39% of the patients with a negative test. Many GPs did not trust a negative rapid test, but were led by the patient’s signs and symptoms in their therapeutic decisions. Thus, 50% of patients with a score ≥ 3 and a negative RADT got an antibiotic.

The Spanish trial [25] and a Swedish study [36] document similar rates of non-adherence to negative RADTs. In Sweden, a negative RADT resulted in a prescription rate of 40% whereas a positive RADT was followed by an antibiotic prescription in about 92% of cases. In the Spanish cRCT 30% of the cases with a negative RADT received an antibiotic and the authors state “The more Centor criteria the patients presented, the greater the number of antibiotics prescribed, regardless of whether RADT was available”.

However, in daily practice, sensitivity (65%) and specificity (85%) of the RADT we used, resulting in a positive predictive value of 46%, were quite disappointing, compared to the manufacturer’s information of a sensitivity of 97.6% and a specificity of 98.4%. A Cochrane review on RADTs in children found a substantial heterogeneity in sensitivity of RADTs across studies with a summary sensitivity of 86% (range 39 – 100%) and a summary specificity of 95% (range 54–100%)[16]. An English HTA-report included 21 point-of-care tests for GAS, among them RADTS and newer tests based on PCR (polymerase chain reaction). The authors report a wide variation in the accuracy of even the same tests between studies [37].

We observed a remarkable heterogeneity of the prescribing behavior across practices with a range of antibiotic prescriptions from 0 to 100% in all three groups. Ultimately, the decision on therapy remained in the GP practice. An observational study reports a similar heterogeneity in antibiotic prescriptions for sore throat [20]. There was also a remarkable heterogeneity concerning the GAS-rate. Overall, the GAS rate of 15% is not much higher than the asymptomatic carriage rate of about 6 – 11%. One has to assume that many patients with a positive throat swab for GAS are likely carriers suffering from a sore throat mainly caused by viruses [19].

The intervention showed clear effects in terms of type of antibiotics prescribed and duration of therapy as well as recommendations of analgesics: GPs in the intervention groups chose penicillin V for 7 days and recommended analgesics more often than the GPs in the control group. These clear, concise parts of the intervention appear to be easier to adhere to.

Overall, we used quite a complex intervention. Only about 1 of 3 patients wanted an antibiotic. But this was strongly associated with an antibiotic prescription (OR 10 in model 1 and OR 12 in model 2). It may have raised antibiotic prescriptions in all groups, indicating little effect of the guideline recommendation to inform the patient about the spontaneous course of a sore throat, and the modest effect of antibiotics [38].

The course of the illness did not differ relevantly between groups confirming the favourable course reported in other studies [1]. GPs may interpret the benign course as a result of their intervention. This confirmation bias might also explain some of the heterogeneity among practices and render in a barrier to any change [39, 40].

### Limitations

HALS was an open cluster-randomized pragmatic trial under real world conditions. The design made blinding impossible. Thus, we cannot exclude selective recruitment of patients. Though using a convenience sample we do not assume relevant limitations to representativeness of our data. By recruiting 520 patients we did not reach the planned number of 570 patients. We decided to randomize practices, in order to avoid contamination. By limiting the number of GPs per practice to two and the number of patients recruited per practice to 11 we tried to reduce at least some cluster effects. However, due to the unexpectedly high heterogeneity in the antibiotic prescription rate among practices the retrospectively calculated ICC of 0.37 was much higher than the ICC of 0.05 that we had assumed. This results in a lack of power. Excluding patients with only light impairment in whom the guideline did not recommend antibiotics might have led to higher prescription rates and smaller effects of the interventions. All practices were instructed in the technique of taking a throat swab, and the practices of the GL-RADT-group to perform the rapid test. However, we cannot rule out the possibility that also untrained practice staff collected swabs or performed the RADTs. Study throat swabs were cultivated and analyzed by the routine procedures described that might have missed some GAS. The GPs almost never used the prepared list for patients not included into the study, but we suspect that they did not recruit consecutively, so that a selection bias of patients can’t be excluded. Time constraints in daily practice are seen as the main reason [14].

With these restrictions, the breadth and quality of the documented data still allowed a comprehensive analysis of the factors influencing the effectiveness of our interventions in daily practice.

## Conclusion

Apparently, neither the implementation of the DEGAM-guideline nor the modified guideline with a targeted rapid test for GAS resulted in fewer antibiotic prescriptions for sore throat.

Application of the Centor or McIsaac score as proposed in the DEGAM-guideline did not lower prescriptions for sore throat. Given the low and heterogeneous prevalence of GAS and the low specificity of the scores in our study, an approach based on scores will apparently overestimate the frequency of GAS pharyngitis by a factor of 3 to 4.

In the rapid test group there were, however, significantly more patients with high scores than in the guideline group. Adjustment resulted in fewer antibiotic prescriptions in the rapid test group compared to the group using only scores, despite GPs’ low adherence to negative RADTs. In view of the scepticism of physicians towards negative test results and the poor quality of the rapid test in daily practice, we suggest to explore the effects of point-of-care tests for GAS in studies focussing on training of practices and adherence of GPs to negative results. This training should put more emphasis than in the present study on information on GAS carrier status, the natural course of a sore throat and the expected benefits and disadvantages from antibiotic treatment.

## Data Availability

The data are available on reasonable request. Please contact the corresponding author. Appropriate data transfer agreements with the participating institutions will be required.

## Abbreviations

GAS: Group A beta-hemolytic streptococci
RADT: Rapid antigen detection test for GAS (Strep-tests)
GPs: General practitioners (general practitioners and specialists in internal medicine working in general practice)
DEGAM: German College of General Practitioners and Family Physicians
RCT: Randomized controlled trial
cRCT: Cluster-randomized, controlled trial
GL-group: Guideline group
GL-RADT- group: Guideline and targeted rapid antigen detection test group
UC-group: Usual care group
PCR: Polymerase chain reaction
